# TRAIL and RIPK3 Do Not Predict Mortality and Acute Kidney Injury in Critically Ill Patients at Admission to the Intensive Care Unit—A Single-Center Cohort Study

**DOI:** 10.3390/ijms26189090

**Published:** 2025-09-18

**Authors:** Katarzyna Kakareko, Alicja Rydzewska-Rosolowska, Irena Glowinska, Elzbieta Regulska, Karolina Rygasiewicz, Ewa Koc-Zorawska, Tomasz Hryszko

**Affiliations:** 1 2nd Department of Nephrology, Hypertension and Internal Medicine with Dialysis Unit, Medical University of Bialystok, 15-089 Bialystok, Poland; 2 Faculty of Pharmacy, University of Castilla-La Mancha, 02008 Albacete, Spain; 3 Faculty of Chemistry, University of Bialystok, 15-089 Bialystok, Poland; 4 Department of Anesthesiology and Intensive Care, Medical University of Bialystok, 15-089 Bialystok, Poland

**Keywords:** TRAIL, RIPK3, TNF-related apoptosis-inducing ligand, receptor-interacting serine/threonine protein kinase-3, ICU stratification, critically ill

## Abstract

TNF-related apoptosis-inducing ligand (TRAIL), a protein involved in apoptosis, and receptor-interacting serine/threonine protein kinase-3 (RIPK3), implicated in necroptosis, are known to change during organ damage and have been studied as prognostic markers in sepsis; however, cell death mechanisms also play a role in other critical illnesses. This study aimed to assess the utility of TRAIL and RIPK3 in predicting mortality and acute kidney injury (AKI) among unselected intensive care unit (ICU) patients. We performed a single-center prospective cohort study including 142 consecutive ICU admissions, collecting blood samples for TRAIL and RIPK3 measurement within 24 h of admission. The primary endpoints were in-hospital mortality and incident AKI during the ICU stay, with additional analysis of mortality over a two-year follow-up. Neither TRAIL nor RIPK3 levels at admission were significantly associated with in-hospital mortality, long-term mortality, or AKI incidence. However, higher RIPK3 levels correlated with shorter time to AKI occurrence. These results suggest that despite earlier evidence supporting their prognostic value in sepsis, TRAIL and RIPK3 did not reliably predict outcomes in a heterogeneous ICU population, underscoring the complexity of critical illness and the need for multiparametric biomarker strategies to improve risk stratification.

## 1. Introduction

Patients admitted to Intensive Care Units (ICU) are at the highest risk of mortality due to severity and complexity of their conditions [1]. Organ disfunction is one of the key components that correlate with higher ICU mortality, which may exceed 20–30% depending on the primary diagnosis and comorbidities [2]. Early recognition of patients at highest risk is essential for optimizing treatment strategies, allocating resources efficiently and intensifying monitoring and therapeutic strategies. However, this remains a persistent clinical challenge. Time is crucial when managing critically ill patients, particularly in the early phase of ICU admission, as timely decisions can significantly impact outcomes. Retrospective studies have reported early mortality rates—defined as death occurring within the first five days of ICU stay—as high as 40%, underscoring the need for rapid and accurate risk stratification immediately upon admission [3]. To assess the extent of organ failure, clinicians commonly use scoring systems such as Sequential Organ Failure Assessment (SOFA) score [4]. It was proven that SOFA is useful not only for regular assessment of organ function but also for predicting short-term patient outcomes and mortality [5], which is important for clinical management. Given the urgent need to rapidly stratify patients based on prognosis, there is continuous interest in identifying novel biomarkers that offer equal or greater predictive power to current scoring systems, while being faster and simpler to implement. Due to these immense expectations, this marker is often referred to as the ‘Holy Grail’.

Among the most promising biomarker candidates are those linked to fundamental pathophysiological pathways, such as regulated cell death and immune dysregulation. Cellular mechanisms underlying critical illness—especially sepsis—include processes such as apoptosis and necroptosis both of which have been implicated in organ injury and failure. TNF-related apoptosis-inducing ligand (TRAIL), a member of the TNF superfamily, is a regulator of cellular response in sepsis. Its role is to ‘recruit’ activated leukocytes to areas of infection to help reduce inflammation [6,7]. Reduced levels of circulating TRAIL have been correlated with higher illness severity and increased mortality in sepsis cohorts [8,9] suggesting that TRAIL could serve as a prognostic biomarker.

In parallel, receptor-interacting serine/threonine protein kinase-3 (RIPK3) is involved in programmed cell death (necroptosis) and is present during organ damage. In vivo studies have demonstrated that RIPK3 enhances kidney tubular injury by promoting oxidative stress and mitochondrial dysfunction [10]. Further pre-clinical studies showed that elevated RIPK3 concentrations are associated with sepsis and with sepsis-induced acute kidney injury (AKI) [10].

Given that apoptosis and necroptosis are fundamental cellular processes occurring in many forms of critical illness—not just sepsis—it remains unclear whether these biomarkers have predictive value in a real-world, heterogeneous ICU population, encompassing various causes of critical illness. The aim of the present study was therefore to systematically evaluate whether early measurements of TRAIL and RIPK3 can predict mortality and AKI in a general ICU cohort. By addressing this gap, we aim to determine the potential utility of these molecular markers in risk stratification and personalized management of critically ill patients.

## 2. Results

### 2.1. Patients’ Characteristics

The baseline characteristics of the study population at the time of ICU admission are presented in Table 1. A total of 142 patients were prospectively enrolled and followed until discharge or occurrence of an endpoint. The median age was 64 years, ranging from 19 to 94 years, and 58 (41%) patients were female. Sepsis was diagnosed in 38 (27%) patients. Other non-infectious reasons for ICU admission included multiple trauma, surgical complications, cardiopulmonary arrest and intracerebral hemorrhage, among others.

Nearly all patients (96%) required invasive mechanical ventilation, with a median duration of 8 days (IQR, 3–16). Incident AKI was diagnosed during hospitalization in 14 (10%) patients with a median time to onset of 2 days (IQR, 1–6). The median length of ICU stay was 15 days (IQR, 5–32). In-hospital mortality was 32% (45 patients), with the median time to death being 64 days (IQR, 48–77). During the two-year follow-up, 89 (63%) patients died and 3 were lost to follow-up.

### 2.2. TRAIL and RIPK3 Plasma Levels and Associations with Clinical Variables

Plasma concentrations of TRAIL and RIPK3 were measured within 24 h of ICU admission. Correlation analyses were performed to assess relationships with inflammatory markers, severity scores, and comorbidities.

**Figure 1:**
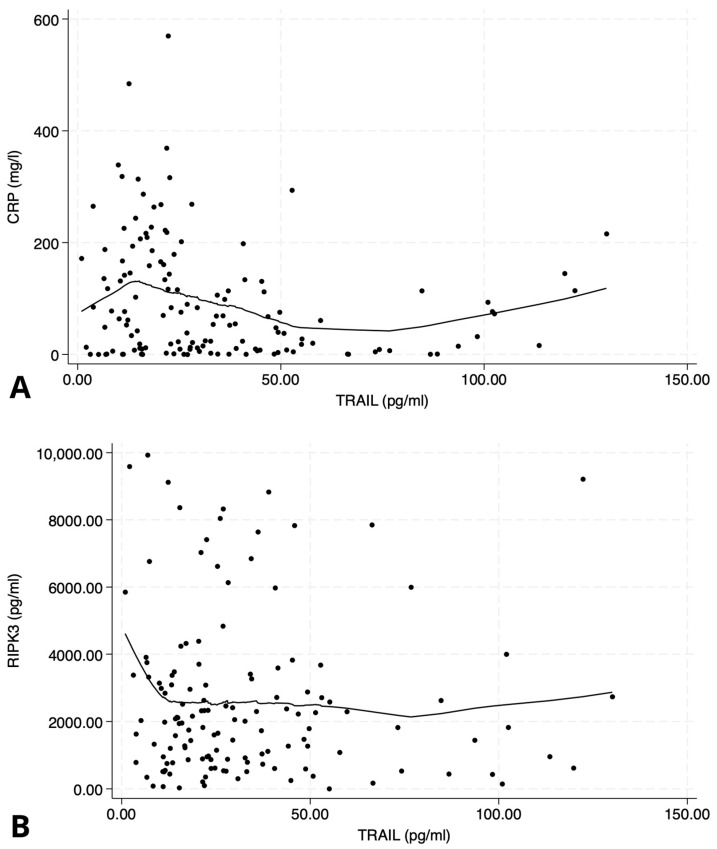
Scatter plots illustrating the relationship between TNF-related apoptosis-inducing ligand (TRAIL) and inflammatory/necroptosis markers. (**A**) Negative correlation between TRAIL and C-reactive protein (CRP); Spearman’s ρ = −0.20, *p* < 0.05. (**B**) No significant correlation between TRAIL and receptor-interacting protein kinase-3 (RIPK3); Spearman’s ρ = 0.04, *p* = 0.7. Data are displayed with LOWESS-smoothed trend lines to highlight potential non-linear patterns.

TRAIL levels demonstrated a significant negative correlation with C-reactive protein (*p* < 0.05, Figure 1A), suggesting an inverse relationship with systemic inflammatory activity. Among recorded comorbidities, TRAIL was associated specifically with a history of ischemic heart disease (*p* = 0.028).

RIPK3 levels showed an association with hypertension (*p* = 0.025). Additionally, TRAIL concentrations correlated negatively with illness severity, as measured by APACHE II and SOFA scores (*p* = 0.04 and *p* = 0.013, respectively).

However, there was no significant correlation observed between TRAIL and RIPK3 concentrations (*p* = 0.7, Figure 1B), suggesting that these biomarkers may reflect distinct biological pathways.

### 2.3. Association of TRAIL and RIPK3 with In-Hospital Mortality

**Figure 2:**
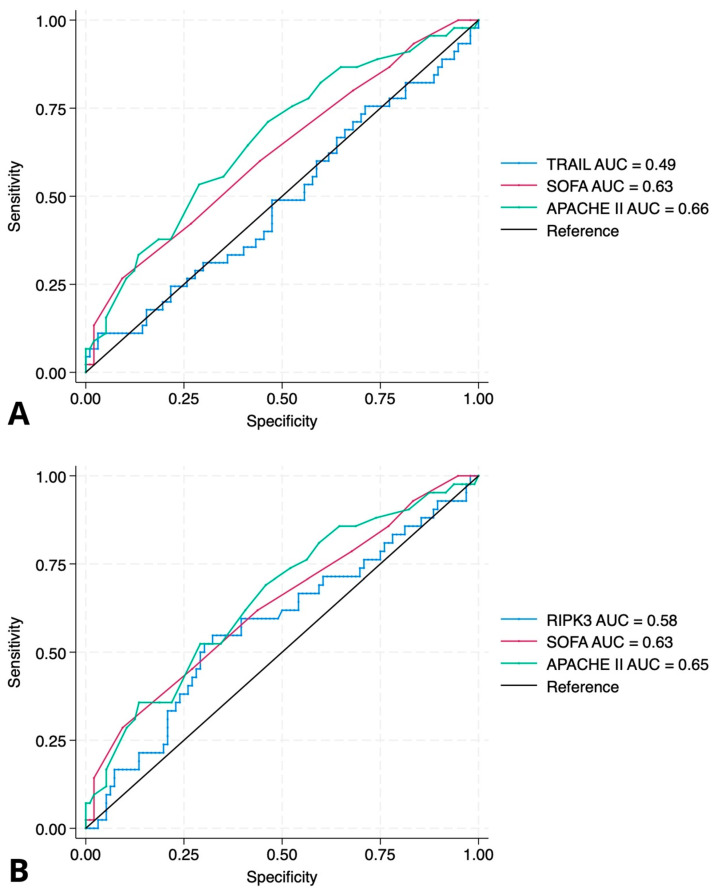
ROC curves for prediction of in-hospital mortality. (**A**) ROC curve for TRAIL compared to SOFA and APACHE II scores. The area under the curve (AUC) for TRAIL was 0.49 (95% CI: −0.011 to 0.015, *p* = 0.75), indicating no prognostic discrimination. In contrast, SOFA and APACHE II scores had AUCs of 0.63 (OR = 1.25, 95% CI: 1.05–1.50; *p* = 0.012) and 0.66 (OR = 1.09, 95% CI: 1.03–1.16; *p* = 0.002), respectively. (**B**) ROC curve for RIPK3 compared to SOFA and APACHE II scores. The AUC for RIPK3 was 0.58 (95% CI: −0.0006 to 0.0019, *p* = 0.33), suggesting limited predictive value. SOFA and APACHE II retained better discriminative ability with AUCs of 0.63 and 0.65. Abbreviations: TRAIL—TNF-related apoptosis-inducing ligand; RIPK3—receptor-interacting protein kinase-3.

To assess the prognostic value of baseline plasma levels of TRAIL and RIPK3 measured at ICU admission, univariate logistic regression analyses were performed. Neither TRAIL (*p* = 0.75) nor RIPK3 (*p* = 0.33) showed a statistically significant association with in-hospital mortality. These findings were further illustrated using ROC curve analysis (Figure 2). TRAIL showed no discriminative ability for in-hospital mortality (AUC = 0.49, 95% CI: −0.011 to 0.015, *p* = 0.75), while RIPK3 also failed to provide meaningful prognostic discrimination (AUC = 0.58, 95% CI: −0.0006 to 0.0019, *p* = 0.33). In contrast, both the SOFA and APACHE II scores showed better predictive performance, with AUCs of 0.63 (SOFA: OR = 1.25, 95% CI: 1.05–1.50, *p* = 0.012) and 0.66 (APACHE II: OR = 1.09, 95% CI: 1.03–1.16, *p* = 0.002), respectively. In subgroup analyses restricted to patients with sepsis (n = 38), neither TRAIL nor RIPK3 plasma concentrations were significantly associated with in-hospital mortality (TRAIL: OR 1.02, 95% CI 0.99–1.05, *p* = 0.19; RIPK3: OR 1.00, 95% CI 0.999–1.0004, *p* = 0.31).

### 2.4. Association of TRAIL and RIPK3 with 2-Year Mortality

We also evaluated the association between biomarker concentrations at ICU admission and all-cause mortality during the two-year follow-up period. Neither TRAIL (*p* = 0.35) nor RIPK3 (*p* = 0.13) concentrations were predictive of mortality in this extended timeframe.

### 2.5. Association of TRAIL and RIPK3 with AKI

Logistic regression analyses were performed to assess the association between admission biomarker concentrations and the development of incident AKI during ICU stay. Neither TRAIL (*p* = 0.5) nor RIPK3 (*p* = 0.8) demonstrated a significant association with the occurrence of AKI.

Interestingly, RIPK3 concentrations exhibited a significant negative correlation with time to AKI onset (*p* = 0.019), indicating that higher baseline levels were associated with earlier development of AKI during hospitalization.

### 2.6. Sensitivity Analyses

To evaluate the robustness of our findings, we conducted sensitivity analyses adjusting for age, sex, and illness severity scores (APACHE II and SOFA) in multivariable logistic regression models. These adjustments did not materially change the results, and the associations between biomarkers and clinical outcomes remained non-significant. To further examine the robustness of our findings, we performed additional sensitivity analyses adjusting for cardiovascular burden and baseline kidney function. Cardiovascular burden was defined as a composite variable including ischemic heart disease, chronic heart failure, and hypertension. Baseline renal function was accounted for using eGFR estimated by the CKD-EPI equation. Multivariable logistic regression models with these additional covariates, as well as age, sex, and SOFA score, did not materially change the results. Neither TRAIL nor RIPK3 emerged as independent predictors of in-hospital mortality or AKI, and the associations remained non-significant. In Cox regression models evaluating time to AKI, both TRAIL and RIPK3 showed no independent association after adjustment for these comorbidities.

## 3. Discussion

In our study, we aimed to evaluate the utility of TRAIL and RIPK3 in critically ill patients admitted to the ICU. We found that a single measurement of these biomarkers at the admission does not predict in-hospital and two-year mortality, nor does it predict incident AKI.

### 3.1. Mortality

TRAIL, a protein primarily expressed by leukocytes, exerts pro-apoptotic effects that contribute to the resolution of inflammation, as demonstrated in vitro [11,12]. Although experimental data demonstrating the link between TRAIL and sepsis remain limited [6,13,14], three clinical studies to date have evaluated its role in septic patients, consistently reporting an inverse association with sepsis severity [8,9,15]. However, findings from these studies concerning in-hospital mortality are inconclusive. While Schenck et al. demonstrated an association with in-hospital mortality in 2 out of 3 ICU cohorts predominantly consisting of sepsis patients [8] and Tian et al. reported similar results in a smaller group of patients [9], another study did not observe an association between TRAIL and 28- and 90-day mortality in ICU patients with sepsis [15]. These heterogeneous results highlight that the prognostic value of TRAIL may depend on patient selection and underlying pathology. Consistent with this variability, our study did not demonstrate an association between TRAIL levels at admission and either in-hospital or two-year mortality. One possible explanation is that only 27% of our patients had sepsis, whereas previous positive studies focused specifically on septic populations. The differences in pathophysiology between sepsis and other critical conditions may influence TRAIL activation, limiting its utility as a universal prognostic marker in an unselected ICU population.

TRAIL showed a negative correlation with CRP and severity scores (APACHE II and SOFA) in our cohort. This is consistent with its role in modulating inflammation and apoptosis [11,16]. Elevated CRP levels indicate heightened systemic inflammatory activity, while lower TRAIL levels may reflect impaired regulation of this response. Conversely, TRAIL is involved in inducing apoptosis in infected or damaged cells, thus playing a critical role in immune surveillance and the resolution of inflammation. However, despite these associations with severity, it did not translate into prognostic significance for mortality. This discrepancy suggests that while TRAIL may reflect current inflammatory status, it may not independently predict long-term outcomes across heterogeneous ICU populations.

Beyond sepsis, the utility of TRAIL as a prognostic marker has also been investigated in other infectious conditions. For example, TRAIL levels have been shown to predict severity and prognosis in patients with community-acquired pneumonia [17] and have been associated with ICU-mortality in patients with ARDS secondary to SARS-CoV-2 infection based on protein profiling of bronchial aspirates [18]. Additionally, TRAIL’s ability to selectively induce apoptosis in tumor cells has led to development of TRAIL-receptor agonist or recombinant TRAIL for use in cancer therapy [19], highlighting its potential not only as a biomarker but also as a therapeutic target to restore immune balance. Experimental models of polymicrobial sepsis in mice have similarly demonstrated improved survival with recombinant TRAIL administration [13].

Due to the expression of TRAIL receptors within the cardiovascular system [20,21], its role has also been explored in cardiomyopathy [22], atherosclerosis [23,24] and pulmonary hypertension [25], with data suggesting potential value in cardiovascular risk stratification [26]. Prospective studies have even demonstrated an inverse association between baseline TRAIL concentrations and all-cause or cardiovascular mortality over a six-year follow-up [27]. One proposed mechanism underlying these protective effects involves TRAIL’s ability to recruit activated leucocytes and induce apoptosis, thereby promoting resolution of inflammation and restoring immune homeostasis [28].

Taken together, these diverse findings underscore that TRAIL’s biological effects are highly context-dependent, influenced by disease-specific mechanisms and patient characteristics. Our results, showing no predictive value for mortality in a heterogeneous ICU population with a low proportion of sepsis cases, align with this complexity and suggest that TRAIL may have limited utility as a universal prognostic marker across all critically ill patients.

One additional aspect to note is that, in our subgroup of septic patients (27% of the cohort), we also did not observe a significant association between either TRAIL or RIPK3 levels and in-hospital mortality. This lack of significance may, at least in part, reflect the limited sample size of this subgroup. Furthermore, methodological differences compared to previous studies should be acknowledged: we analyzed biomarker levels as continuous variables contrary to prior studies [8,14]. Given our cohort size, we deliberately refrained from additional stratification, which may partly explain the divergence from earlier reports.

Necroptosis, mediated by RIPK3, is a form of regulated, non-apoptotic cell death implicated in the pathogenesis of critical illness. Flow cytometry analyses have demonstrated significantly increased intracellular RIPK3 expression in sepsis patients compared with healthy controls [29]. Experimental data have also implicated RIPK3 in the development of ventilator-induced lung injury in murine models [30]. In contrast to these findings, our study did not observe an association between RIPK3 levels at ICU admission and mortality or organ dysfunction. One potential explanation for this discrepancy lies in differences in study design and patient populations. For instance, in the study by Ma et al. [31], baseline blood samples for one-third of the cohort were collected up to 48 h after ICU admission, potentially capturing different phases of critical illness compared to our protocol of sampling within 24 h. Moreover, approximately 75% of Ma et al.’s cohort [31] comprised patients with sepsis or septic shock, whereas only 27% of our study population met sepsis criteria, reflecting differences in underlying disease mechanisms that may influence RIPK3 activation.

In our cohort, the overall two-year mortality was 63%. Among the 97 patients who survived hospitalization, 44 died during the follow-up period and 3 were lost to follow-up, corresponding to a two-year post-discharge mortality of 44%. This value is somewhat higher but still comparable to previously reported long-term mortality rates among ICU survivors, which range from 20% to over 38% depending on patient age, comorbidities, and length of ICU stay [32,33,34,35]. Our analysis of two-year mortality should therefore be interpreted as exploratory. Predicting long-term outcomes based solely on early ICU biomarkers is inherently challenging, as survival after ICU discharge is shaped by a complex interplay of post-acute factors. Because we only had access to vital status but not to specific causes of death, these important determinants could not be accounted for, which limits the interpretation of our findings.

### 3.2. AKI

Observations from experimental AKI models have been translated into human clinical trials. RIPK3 was reported to be associated with AKI in critically ill trauma patients, but the association was observed only after 48 h [36]. In our cohort, we studied the entire ICU population and collected blood samples early upon admission, which might explain why we did not detect changes. This single time-point measurement was intentional, as early-phase biomarkers are often considered most clinically useful given the high early mortality rate and the critical need for rapid risk stratification in the ICU. The observed negative correlation of RIPK3 with the time to AKI diagnosis supports this theory. The lower RIPK3 concentrations were associated with a longer time to develop AKI. The lack of association between baseline biomarker levels and clinical outcomes may, in part, reflect the dynamic nature of critical illness. Moreover, our aim was to determine if TRAIL or RIPK3 measured at admission predicted AKI at any point during hospitalization; we did not measure RIPK3 concentration during an AKI episode. We also acknowledge that the number of AKI cases in our study was small, and differences in AKI etiology may have influenced the result.

For TRAIL, we found no association with AKI development. This may be due to its primary role in immune regulation and apoptosis, which may not directly reflect the mechanisms driving AKI in critically ill patients.

In our study, neither TRAIL nor RIPK3 showed a direct association with AKI when adjusted for age, sex, disease severity (SOFA), sepsis, cardiovascular burden, and baseline eGFR. Given the limited number of AKI events, the statistical power to detect independent associations was low, and the results should be interpreted cautiously. Nevertheless, the trend observed for TRAIL (HR ~1.5) may suggest an indirect contribution through comorbid conditions. Cardiovascular burden, defined as the coexistence of ischemic heart disease, congestive heart failure, and hypertension, is closely linked with both AKI and mortality and may influence cell signaling pathways in which TRAIL and RIPK3 are involved. Taken together, our findings support the notion that TRAIL and RIPK3 do not act as independent predictors of AKI or mortality, but rather as components of a broader network of pathways influenced by underlying cardiovascular and renal dysfunction.

### 3.3. Future Directions and Potential Applications

Our findings underscore the complexity of critical illness and the challenges of developing universal biomarkers for prognostication in the ICU. Although single biomarkers such as TRAIL and RIPK3 may not reliably predict mortality or AKI across heterogeneous patient populations, they remain biologically relevant indicators of apoptosis and necroptosis. Future research should explore multimarker panels that integrate complementary pathways, serial measurements to capture dynamic changes over time, and machine learning approaches to improve predictive accuracy. Additionally, understanding the mechanistic roles of TRAIL and RIPK3 in organ dysfunction could identify new therapeutic targets aimed at modulating these cell death pathways to mitigate organ damage in critically ill patients. Although our findings did not support the prognostic utility of TRAIL and RIPK3 in isolation, these markers may still provide value in biological phenotyping of critically ill patients. Identifying subgroups characterized by heightened apoptotic or necroptotic activity could aid in stratifying patients for targeted interventions, such as immunomodulatory therapies. This concept aligns with prior work demonstrating that immunologic phenotyping can help guide host-directed therapies in sepsis [37,38].

Importantly, negative findings such as ours play a critical role in refining the direction of translational research. Biomarker studies are prone to publication bias, with positive results more likely to be reported. By transparently presenting null findings from a well-characterized cohort, our study contributes to a more balanced understanding of biomarker performance and helps prevent overestimation of clinical utility in heterogeneous populations.

While these results offer valuable insights, they should be interpreted in the context of several limitations. First, this was a single-center study which may restrict the generalizability of our findings to other settings and patient populations. Second, biomarker concentrations were measured only once, within 24 h of ICU admission—limiting our ability to capture dynamic changes over time. Serial measurements could have provided additional insights into temporal dynamics and might better capture prognostic value, particularly for markers like RIPK3 whose association with AKI has been reported at later time points. Lastly, the relatively small number of AKI events in our cohort could have limited the statistical power to detect more subtle relationships.

Despite these limitations, our study has several important strengths. We enrolled a well-characterized, unselected ICU cohort and conducted a long-term follow-up, including two-year mortality data. The relatively high number of observed clinical events enhances the reliability of our findings and reinforces the relevance of our conclusions for real-world ICU practice.

In conclusion, while TRAIL and RIPK3 are biologically plausible markers reflecting apoptosis and necroptosis, our study did not demonstrate their prognostic significance for mortality or incident AKI in a heterogeneous ICU population. These findings clarify the limitations of broadly applying sepsis-focused biomarker evidence and suggest it may be overly optimistic to expect that a single marker can reliably predict outcomes across all critically ill patients. Instead, they emphasize the need for multiparametric strategies, including panels of complementary biomarkers and serial assessments, to better capture the complex and dynamic pathophysiology of critical illness. Although TRAIL and RIPK3 are not yet suitable for routine clinical use, the search for more effective prognostic tools—the so-called ‘Holy Grail’—remains essential.

## 4. Materials and Methods

### 4.1. Study Population

The present study is a secondary analysis from a previously published single-center cohort study conducted on consecutive patients admitted to the ICU [39]. Critically ill patients admitted to the ICU were considered eligible for inclusion unless they were under 18-years old, on dialysis or pregnant. Additionally, exclusion criteria included also serum creatinine concentration higher than 1.5 mg/dL at the time of admission, as one of the aims of the study was to focus on AKI, and patients with a previous history of chronic kidney disease could make the analysis harder to interpret.

The study protocol was approved by the local ethics committee (approval no. APK.002.29.2020) and adhered to the principles of the Declaration of Helsinki. Written informed consent was obtained from all patients or their legally authorized representatives prior to enrollment.

### 4.2. Design of the Study

This study followed the Strengthening the Reporting of Observational Studies in Epidemiology (STROBE) guidelines for reporting observational studies. Upon admission physiological parameters, including the Sequential Organ Failure Assessment (SOFA) score and Acute Physiology and Chronic Health Evaluation II (APACHE II) score, as well as the presence of sepsis (according to the International Sepsis Definitions) were obtained for each patient. Within 24 h from the admission to the ICU (baseline) blood samples for measurements TRAIL and RIPK3 were collected, centrifuged and aliquoted. Whenever feasible, sampling was performed during the first hours after ICU admission. In most cases this corresponded to the initial blood draw, although the exact timing could vary depending on the circumstances of admission and clinical workflow. Tests for other parameters were carried out at the local medical laboratory. Plasma samples for TRAIL and RIPK3 analysis were frozen at −70 °C until further analysis, without repeated freeze–thaw cycles. Biomarker assays were performed within five years of sample collection. During storage, all samples were maintained under controlled −70 °C conditions with continuous monitoring and automatic warning alerts to ensure sample integrity. Previous studies have demonstrated that protein biomarkers remain stable for extended periods under such conditions, supporting the reliability of our measurements [40,41]. Patients were then followed until the end of their hospitalization. The primary endpoints were incident AKI (defined according to the Kidney Disease Improving Global Outcomes Work Group guidelines) and in-hospital mortality during the ICU stay. The secondary outcome was 2-year mortality.

### 4.3. Measurement of Plasma TRAIL and RIPK3

Plasma TRAIL and RIPK3 were measured from stored aliquots using commercially available enzyme-linked immunosorbent assay (ELISA) kits according to the manufacturer’s recommendations (Human TRAIL/TNFSF10 Quantikine ELISA Kit, R&D Systems, Minneapolis, MN, USA and Human Receptor-Interacting Serine/Threonine-Protein Kinase 3 (RIPK3) ELISA Kit, Mybiosource Inc., San Diego, CA, USA, respectively).

Plasma TRAIL and RIPK3 were index tests and SOFA and APACHE II scales were used as references (reference standard according to STARD guidelines).

### 4.4. Two-Year Follow-Up

The long-term outcome of the study cohort was retrieved from a national death registry database provided by the Polish Ministry of Digital Affairs. This registry allowed us to track the vital status of participants over the two-year period following the initial study. We were able to obtain information on the occurrence of death for each participant, but the database did not provide specific causes of death.

### 4.5. Statistical Analysis

Continuous variables are presented as median and interquartile range (IQR) and were compared between survivors and non-survivors with t test and Mann–Whitney test. Categorical variables are reported as frequency and percentage and were compared using Chi-square test. Both Pearson and Spearman correlation coefficients were calculated depending on the distribution and linearity of the data. The prognostic significance of TRAIL and RIPK3 was evaluated with uni- and multivariate logistic regression analysis. Receiver operating characteristic (ROC) curve analysis was performed to assess the discriminative ability of TRAIL, RIPK3, SOFA, and APACHE II scores in predicting in-hospital mortality. Patients with missing data were excluded from the analysis (4 patients were excluded from statistical analysis for RIPK3 as data on their RIPK3 level were missing due to results above the assay range).

All tests were two-sided, and significance was accepted at *p* < 0.05. All statistical computations were performed with R ver. 4.3.0 (Docker Hub, Vienna, Austria).

## Figures and Tables

**Table 1:** Baseline characteristics of the study population.

Characteristic	All	Survivors	Non–Survivors	*p* Value
	(n = 142)	(n = 97)	(n = 45)	
**Demographics**				
**Age years median (IQR)**	64 (48–77)	61 (46–71)	69 (53–81)	*p* = 0.03 *
**Sex (M/F), n (%)**	84/58 (59/41)	62/35 (64/36)	22/23 (49/51)	*p* = 0.09
**Incident AKI, n (%)**	14 (10%)	6 (6)	8 (18)	*p* = 0.03 *
**Comorbidities, n (%)**				
**Diabetes mellitus**	21 (15)	12 (12)	9 (20)	*p* = 0.2
**Chronic heart failure**	45 (32)	28 (29)	17 (38)	*p* = 0.2
**Ischemic heart disease**	27 (19)	17 (18)	10 (22)	*p* = 0.5
**Hypertension**	63 (44)	39 (40)	24 (53)	*p* = 0.1
**COPD**	18 (13)	12 (12)	6 (13)	*p* = 0.8
**Sepsis**	38 (27)	20 (21)	18 (40)	*p* = 0.01 *
**Illness severity, median (IQR)**				
**APACHE II**	18 (12–22)	17 (11–21)	21 (17–26)	*p* < 0.01 *
**SOFA**	9 (8–11)	9 (8–11)	10 (9–12)	*p* < 0.01 *
**Laboratory, median (IQR)**				
**Hemoglobin, g/dL**	11.75 (10.2–13.1)	12.1 (10.2–13.1)	11.2 (10.1–13.0)	*p* = 0.6
**Creatinine, mg/dL**	0.82 (0.62–1.06)	0.82 (0.61–1.04)	0.86 (0.64–1.13)	*p* = 0.4
**CRP, mg/L**	61.8 (10–145)	49 (8.3–131.0)	87.5 (20.15–195)	*p* = 0.07
**Procalcitonin, ng/mL**	0.54 (0.14–2.81)	0.44 (0.12–1.9)	0.71 (0.17–4.31)	*p* = 0.6
**TRAIL, pg/mL**	24.58 (14.61–43.69)	24.69 (14.61–43.69)	22.6 (15.36–41.39)	*p* = 0.7
**RIPK3, pg/mL**	2022 (876.4–3384)	1829 (836.6–3091.5)	2610.5 (957.5–3681)	*p* = 0.3

Abbreviations: APACHE II—Acute Physiology and Chronic Health Evaluation II; COPD—chronic obstructive pulmonary disease; CRP—C-reactive protein; F—female; IQR—interquartile range; M—male; RIPK3—Receptor-interacting serine/threonine protein kinase-3; SOFA—Sequential Organ Failure Assessment; TRAIL—TNF-related apoptosis-inducing Ligand; * statistically significant.

## Data Availability

Data is contained within the article.

## Abbreviations

The following abbreviations are used in this manuscript:

AKI	Acute kidney injury
APACHE II	Acute Physiology and Chronic Health Evaluation II
COPD	Chronic obstructive pulmonary disease
CRP	C-reactive protein
ELISA	Enzyme-linked immunosorbent assay
ICU	Intensive care unit
IQR	Interquartile range
RIPK3	Receptor-interacting serine/threonine protein kinase-3
SOFA	Sequential Organ Failure Assessment
TNF	Tumor necrosis factor
TRAIL	TNF-related apoptosis-inducing ligand
